# Full-thickness endoscopic myotomy for management of a twisted gastric conduit postesophagectomy

**DOI:** 10.1016/j.igie.2025.12.005

**Published:** 2025-12-24

**Authors:** Shirley Jiang, Ali Dashti, Cynthuja Thilakanathan, John Yee, Roberto Trasolini

**Affiliations:** 1 Department of Medicine, University of British Columbia, Vancouver, Canada; 2 Department of Surgery, University of British Columbia, Vancouver, Canada; 3 Vancouver General Hospital, University of British Columbia, Vancouver, Canada

Gastric conduit dysfunction postesophagectomy occurs in 15% to 30% of patients and can lead to significant morbidity and mortality. Conventional treatments are directed toward managing the increased pyloric tone, including dilation and gastric peroral endoscopic myotomy. However, a minority of refractory patients may have a secondary mechanism.^1^^,^^2^ Herein, we describe a case of gastric conduit dysfunction secondary to helical stenosis managed with endoscopic full-thickness gastric myotomy.

A 69-year-old male underwent an Ivor Lewis esophagectomy at an outside hospital, complicated by significant postoperative dysphagia and regurgitation of liquids and solids, requiring complete jejunostomy feeding. Over several months, he underwent conventional treatment with minimal improvement: pyloric botox injection, endoscopic balloon dilatation of the pylorus to 20 mm, laparoscopic crural revision, and laparotomy with enlargement of the diaphragmatic hiatus and surgical pyloromyotomy. The gastroesophageal anastomosis and pylorus were widely patent on endoscopy. After exhausting prior treatments, he was referred to a therapeutic endoscopist.

Upon review, barium swallow and gastroscopy demonstrated obstruction at a segment of stenosis from twisting of the gastric conduit. This was anatomically analogous to helical stenosis following laparoscopic sleeve gastrectomy, in which twisting of the gastric sleeve leads to obstruction. For gastric sleeve helical stenosis, peroral endoscopic myotomy can be used for tunneled stricturoplasty and effectively remodel the stricture, so a similar approach was undertaken in this case.^3^

After informed consent, endoscopic examination was performed, identifying 2 cm of stenosis from twisting of the gastric conduit (Video 1 and 2, available online at www.igiejournal.org). The access site was chosen 7 cm proximal to the stenosis. A longitudinal mucosotomy was created for entry, followed by subsequent tunneling in the submucosal space with serial injection of methylene blue with saline solution and dissection with an electrosurgical knife. Twisting of the muscular plane was noted in the segment of stenosis, corresponding with luminal obstruction; full-thickness myotomy was completed in this 3-cm segment of stenosis. After myotomy, there was a notable release in the previously stenotic segment. Mucosotomy was closed with through-the-scope clips (Fig. 1).

**Figure 1:**
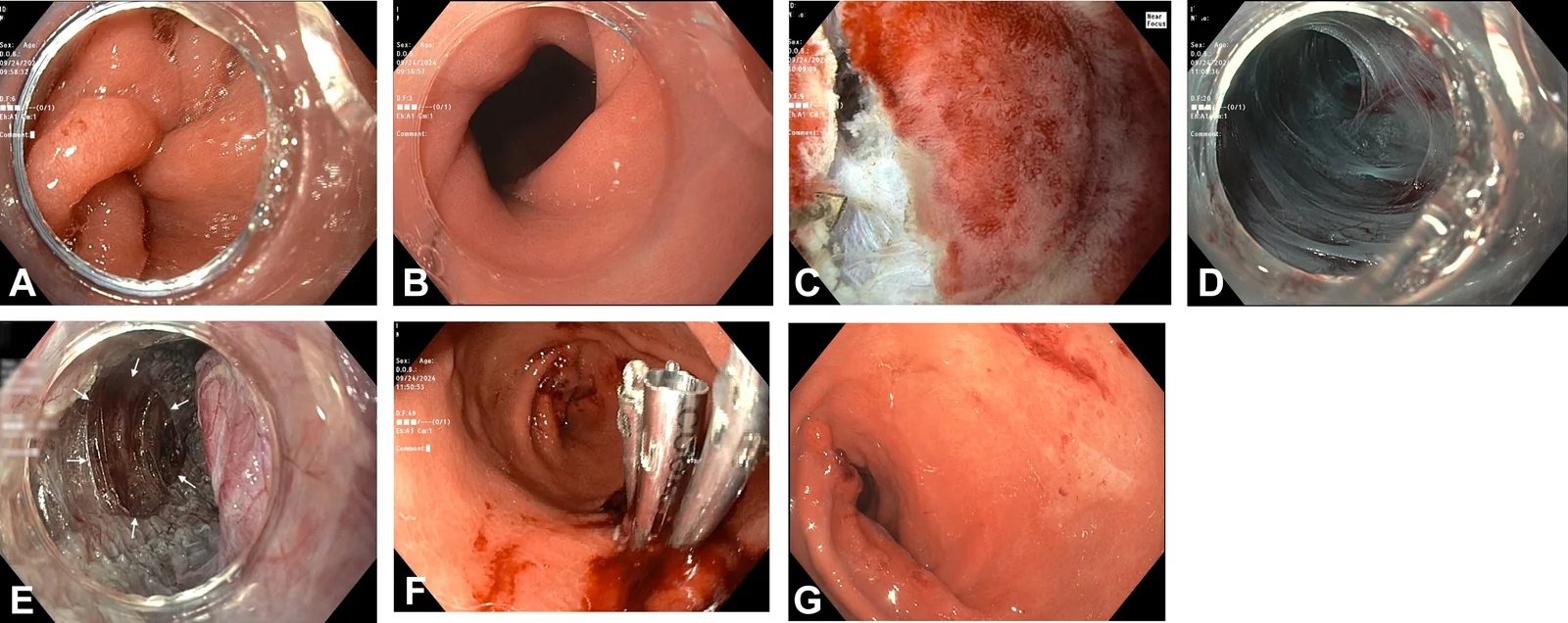
Management of a twisted gastric conduit with full-thickness peroral endoscopic myotomy. **A,** Stenosis of the twisted gastric conduit proximal to the pylorus. **B,** Lumen of the stenosis. **C,** Creation of mucosotomy. **D,** Submucosal tunnel with visible tortuosity within tunnel and associated resistance to endoscope passage in this area. **E,** Full-thickness myotomy with exposure of the serosal layer (*arrows* pointing to serosa). **F,** Mucosotomy closed with through-the-scope clips. **G,** Final image of lumen following full-thickness myotomy.

Two months following full-thickness endoscopic myotomy for the stenosis from twisting of the gastric conduit, the patient could consume solid meals and discontinued jejunostomy feeding. Postprocedural barium swallow showed oral contrast passing through the previously stenotic segment (Fig. 2). Repeat endoscopy in 5 and 8 months demonstrated ongoing improvement in stenosis.

**Figure 2:**
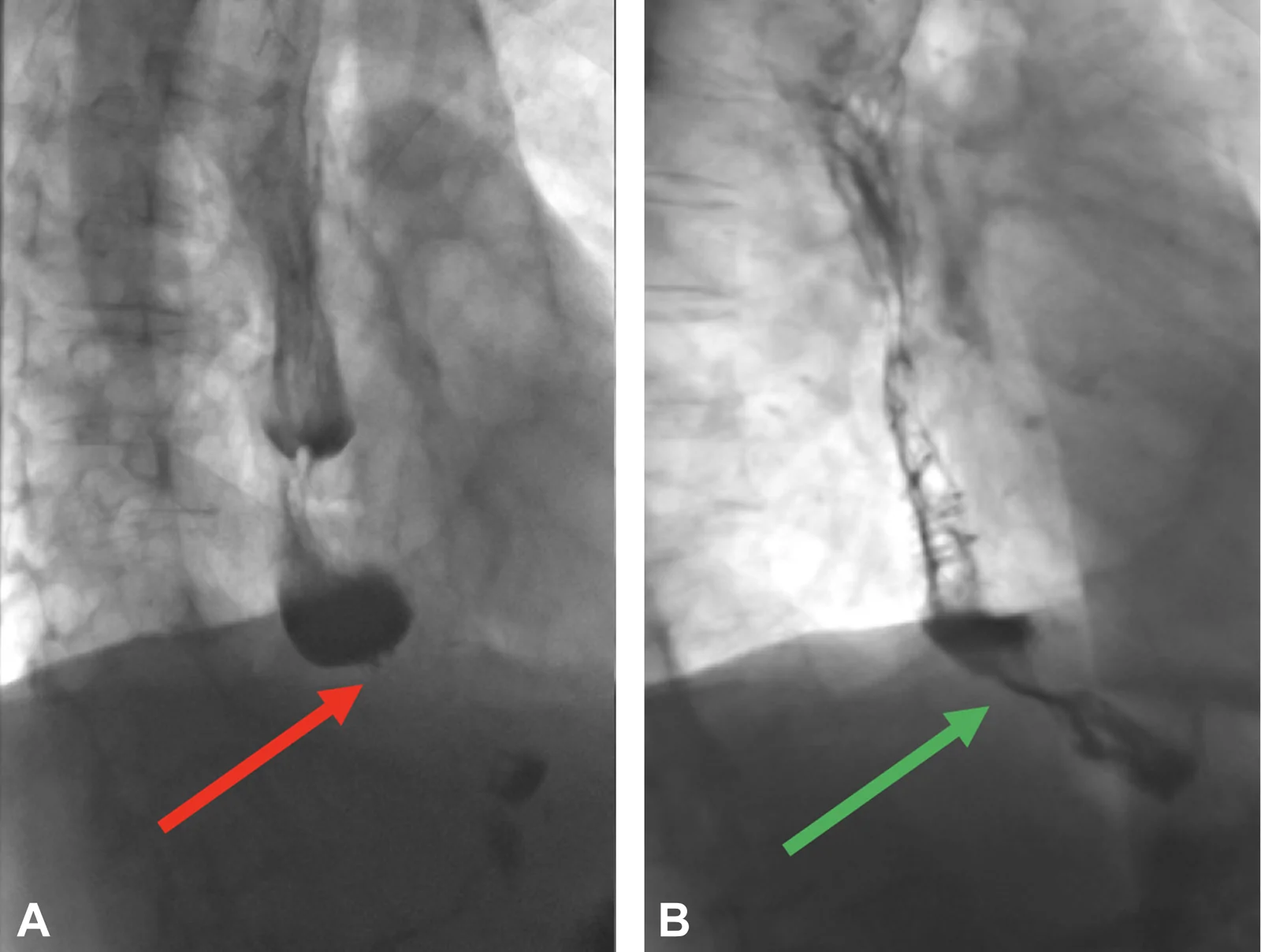
Preprocedural and postprocedural barium swallow. **A**, Preprocedural barium swallow with hold up of contrast at the stenosis (*red arrow*). **B**, Postprocedural barium swallow with passage of contrast (*green arrow*).

Endoscopic myotomy was a safe and effective treatment in this case of stenosis from a twisted gastric conduit postesophagectomy. With advances in third-space endoscopy, surgery is no longer the only or the optimal treatment for stenosis in selected patients.

## Patient consent

Patient consent was obtained for this article.

## Disclosure

The following author disclosed financial relationships: R. Trasolini: Consultant and speaker for Boston Scientific and Cook Medical; consultant for Fractyl Health; speaker and principal investigator for clinical trial for Medtronic. All other authors disclosed no financial relationships.

## Supplementary data

Video 1 Full-thickness endoscopic myotomy for management of a twisted gastric conduit postesophagectomy, parts 1 and 2.
